# Quality assurance of data collection in the multi-site community randomized trial and prevalence survey of the children’s healthy living program

**DOI:** 10.1186/s13104-016-2212-2

**Published:** 2016-09-02

**Authors:** Ashley Yamanaka, Marie Kainoa Fialkowski, Lynne Wilkens, Fenfang Li, Reynolette Ettienne, Travis Fleming, Julianne Power, Jonathan Deenik, Patricia Coleman, Rachael Leon Guerrero, Rachel Novotny

**Affiliations:** 1 University of Hawai‘i at Mānoa, Honolulu, HI USA; 2 American Samoa Community College, Mesepa, AS USA; 3 University of Alaska, Fairbanks, AK USA; 4 Northern Marianas College, Saipan, MP USA; 5 University of Guam, Mangilao, GU USA

**Keywords:** Quality assurance, Multi-site, Prevalence, Pacific, Childhood, Obesity

## Abstract

**Background:**

Quality assurance plays an important role in research by assuring data integrity, and thus, valid study results. We aim to describe and share the results of the quality assurance process used to guide the data collection process in a multi-site childhood obesity prevalence study and intervention trial across the US Affiliated Pacific Region.

**Methods:**

Quality assurance assessments following a standardized protocol were conducted by one assessor in every participating site. Results were summarized to examine and align the implementation of protocol procedures across diverse settings.

**Results:**

Data collection protocols focused on food and physical activity were adhered to closely; however, protocols for handling completed forms and ensuring data security showed more variability.

**Conclusions:**

Quality assurance protocols are common in the clinical literature but are limited in multi-site community-based studies, especially in underserved populations. The reduction in the number of QA problems found in the second as compared to the first data collection periods for the intervention study attest to the value of this assessment. This paper can serve as a reference for similar studies wishing to implement quality assurance protocols of the data collection process to preserve data integrity and enhance the validity of study findings.

*Trial registration*: NIH clinical trial #NCT01881373

## Background

Data quality is a key priority when planning a study to guarantee appropriate results and conclusions [1]. Detection and remediation of errors in the data collection process, whether they are made intentionally or not, promotes data integrity. This paper will describe and share the results of a quality assurance (QA) process used in a multi-site childhood obesity prevalence study and intervention trial across the US Affiliated Pacific (USAP) region.

QA is one approach to ensure the validity of study results and preserve data integrity during the data collection process [2]. QA plays an important role in the conduct of the research study by helping to ensure findings and conclusions are correct and justifiable [1]. QA is a process used to prevent problems in the data collection process and to support subsequent data quality [3].

The first step to QA is developing a well-written, comprehensive and detailed procedure manual for data collection [3]. Poorly written manuals increase the chances of errors and risk the validity of the study [2]. Second is developing a rigorous and detailed recruitment and training plan to enforce the value of collecting accurate data. The final step is to monitor and evaluate the process in the field and identify areas of improvement to strengthen the study’s protocol.

The available literature on QA focuses mostly on maximizing the quality of the data, standardizing protocols, personnel training, and data management systems of clinical trials [4–13]. For example, The National Drug Abuse Treatment Clinical Trials Network examined the effect QA had on procedures and outcomes of clinical trials in substance abuse treatment programs [14]. The authors noted the need for a community-based and coordinated system of comprehensive services to ensure integrity of the data collected. This drug abuse treatment study developed a monitoring system accessible to multiple sites that was efficient to meet the needs of each involved treatment program and of the research staff for a multi-level, systematic approach [14]. The intensity of QA monitoring was based on the particular trials, the risks of the interventions, and the experience of the staff with clinical research. Training sessions and the development of flexible tools were administered at each site [14].

QA programs are also beneficial to community-based research studies. The goal of QA is to provide valid and reliable outcomes of a study [15], which can be applied to multi-site studies of any kind. The Girls Health Enrichment multi-site studies (GEMS) program addresses the needs of African–American girls through the development and evaluation of culturally appropriate obesity prevention approaches [16]. The study was conducted at four sites across the nation among similar study populations. A QA procedure was used to ensure the integrity of the data for data management, eligibility violations, procedural errors, concordance in the replicate evaluations, procedures not performed, field-center comparisons, and consistency among the variables [17]. A multicenter, randomized clinical trial called the Dietary Approaches to Stop Hypertension (DASH)-Sodium study also used a QA process to ensure quality in procedures for screening participants, diet preparation, delivery of data collection, staff training, and monitoring activities in several clinical sites [18].

The USAP region, which includes the states of Hawai‘i and Alaska, the US territories of Guam and American Samoa, the US Commonwealth of the Northern Mariana Islands (CNMI), and the Freely Associated States of Micronesia (FAS: States of the Federated States of Micronesia [Pohnpei, Chuuk, Yap, and Kosrae], the Republic of the Marshall Islands, and the Republic of Palau), participated in its first multi-site childhood obesity prevalence and community randomized intervention trial [19]. The USAP region is typically underserved and underrepresented in national, comprehensive nutrition and health research studies. These jurisdictions do not have national nutrition monitoring, such as the National Health and Examination Survey, for nutrition-related health prevention [20]. Limited data on diet, physical activity, obesity, and other health-related indicators within this region restricts the capacity for understanding the care and action needed to control the epidemic of non-communicable chronic diseases present in the region [21]. The need for nutrition monitoring capacity is substantial because of the deficiency of healthcare resources, limited access to primary medical care physicians, high infant mortality rates and poverty levels [22, 23]; yet these same factors challenge the collection of data.

The purpose of this paper is to describe the QA process developed for detection of procedural and data errors for the Children’s Healthy Living (CHL) Program multi-site trial in an underserved region [19]. In addition to describing the process, common findings will be shared as well as strategies used to correct errors. The QA model may serve as a template for other complex multi-site studies in underserved and isolated communities.

## Methods

### Study design

The CHL program included prevalence and intervention studies conducted in the USAP region. The multi-jurisdiction prevalence study and community randomized intervention trial (NIH clinical trial #NCT01881373 [clinicaltrials.gov]) were conducted between Fall 2012 and Spring 2015. CHL’s mission is to elevate the capacity of the region to build and sustain a healthy food and physical environment to help maintain healthy weight and prevent obesity among young children in the Pacific region.

The FAS countries listed previously, participated in the prevalence survey only while Alaska, American Samoa, CNMI, Guam, and Hawai‘i also implemented the community-randomized environmental intervention to prevent childhood obesity with a baseline and 24-month follow-up. CHL focuses on the following six behavioral targets: increase sleep, moderate to vigorous physical activity, fruit and vegetable intake, and water intake, and decrease sedentary behavior and sweetened beverage intake among the communities of the participating jurisdictions [24]. Similar data collection instruments and procedures were used for the prevalence and intervention studies.

Collection of the participant-based measures included anthropometry, diet, physical activity, sleep and Acanthosis Nigricans (present or absent on the back of the neck), in all study locations across the region [24]. Multiple day trainings on collecting all measures, including a standardization process for anthropometric data [25], were implemented in all jurisdictions prior to data collection. For further detail on the study design and protocol, please refer to Wilken et al. [24]. Collection of community-based measures of the environment (schools, stores, parks, churches, etc.) and jurisdiction-based measures (of food prices in food stores, etc.) were also done but were not covered by the same QA methodology and are not reported in this paper.

Institutional Review Board (IRB) approval from the University of Hawai‘i, the University of Guam, the University of Alaska Fairbanks, and the Republic of Palau was granted for the CHL study. Other jurisdictions ceded IRB authority to the University of Hawai‘i. Additional IRB approval was not needed for the QA program, as no additional individual data was collected.

### Quality assurance process

#### Prevention

Standard procedures were used to ensure accurate and consistent measurements throughout the study. Standardized training manuals were developed to document measurement protocols, detail procedures, and minimize errors. The detailed procedures related to anthropometry are reported by Li et al. [25]. Training for Acanthosis Nigricans detection included instructions and a photo array developed by an experienced pediatrician. Staff for each jurisdiction were hired and trained to conduct recruitment, measurement, and data collection for the CHL program. The staff participated in the QA training during scheduled dates. The training reviewed all data collection components and consisted of standardized measurements for anthropometric data to minimize error. The training held before the follow-up measurements in the intervention studies also included a mock data collection session.

#### Detection

The CHL Coordinating Center worked in coordination with each jurisdiction’s team to conduct an overall QA assessment at one point during each measurement collection period—one time in the FAS Region and twice (baseline and follow-up) in Alaska, American Samoa, CNMI, Guam, and Hawai‘i. The goal was to schedule the QA as close to the beginning of the data collection as possible to ensure that any errors would be corrected prior to the majority of the data being collected. QA across all jurisdictions was conducted by the CHL QA Lead (MKF). This individual was from the coordinating center of CHL (Assistant Program Director) whose role was to coordinate overall CHL Program activities. The CHL QA Lead also served as the co-lead of CHL measurement training and standardization and was therefore familiar with data collection processes and protocols.

The QA site visit process centered on measurement-related activities. There were two parts to the QA site visit process: (1) observation in the field and (2) observation of the organization and quality of data in the office. Specific details of each part of the QA process are outlined in Table 1. For each domain, each jurisdiction was assessed for whether all procedures and protocols outlined are met (yes) or whether any of the procedures and protocols are not met (no).

**Table 1:** Children’s Healthy Living (CHL) program “in the office” and “in the field” quality assurance (QA) process

*In the office*
Forms are filed/stored correctly ● All forms are in locked cabinets behind a locked door ● Consent/assent forms are stored separately from other forms ○ ID placed on consent page at the office for filing ● Registration forms stored separately from other forms
Consent and assent form for each child enrolled
Accelerometers are in-house except those logged out ● Observe organizational system for accelerometer downloading and preparation for field
Observe at least three accelerometer downloads and resets (serial numbers noted)
Review at least three forms and logs at random for problems, for example: ● Places in the food and activity log that were not probed ● The data reported in the forms does not make sense (e.g., Birth date of child doesn’t match project age range) ● Blanks in forms that were not initialed/reviewed by CHL staff
*In the field*
Observe field collection set-up ● Check-in station: prepare form packets in advance for easy distribution ● Anthropometry station: calibrate equipment following the CHL protocol
Ensure that proper procedures are being followed at each station in a measurement session a. Orientation/check-in ○ Ensure screening, consent, and photo release forms are completed prior to enrollment ○ Parents/caregiver are provided a copy of the consent form and participant guide ○ Tracking log completed for each child enrolled b. Food and activity log/accelerometer instruction station ○ Food and activity log instruction conducted with food models and tools ○ Food and activity log tips provided with special attention to priority items ○ Measuring cups/spoons and ID food label bags are distributed to parents/caregivers ○ Parents/caregivers are instructed on food and activity logs/accelerometer recording ○ If applicable, parents instructed to check on accelerometers daily and on how to replace bands if needed c. Anthropometry/Acanthosis Nigricans station ○ Child assented prior to data collection (younger than age 7 with CHL staff initials on form while older than age 7 with child initials on form) ○ Team following CHL protocols for anthropometry measurement and Acanthosis Nigricans assessment ○ ID labels, dates and staff initials on data collection forms ○ Anthropometry equipment sanitized regularly ○ Any positive Acanthosis Nigricans screens verified with screening scale and another staff ○ Referral letter process initiated for positive Acanthosis Nigricans screen (e.g., yellow post-it flag placed on form to flag check-out staff to complete referral) ○ Verify anthropometry/Acanthosis Nigricans forms are complete d. Accelerometer placement station ○ Child assented prior to data collection (younger than age 7 with CHL staff initials on form while older than age 7 with child initials on form) ○ Following protocols for placement of accelerometer on child’s non-dominant wrist ○ Verify accelerometer forms are complete e. Forms station ○ Staff available to assist parents/caregivers in completing forms ○ Adequate instruction provided, following question-by-question specifications, at onset and when parents/caregivers requested ○ Forms reviewed and coding instructions followed by CHL staff prior to parent’s departure ○ Verify all forms are completed f. Check out station ○ Check completion of forms ○ Provide completed Acanthosis Nigricans referral to parents/caregivers, if needed ○ Reminders for next visit, if needed ○ Provide compensation ○ Tracking log completed
Ensure that proper procedures are being followed for transport of forms and data ● Lockable repositories used for transportation ● All forms with protected health information transported separately from other forms ● Verify all data transported directly back to office after collection

#### Correction

The QA process concluded with a team debriefing of the measurement activity to review results, discuss corrections and provide clarifications. A QA report was generated to summarize findings across CHL. The QA report was submitted to the IRB to ensure oversight across the jurisdictions. Quality control procedures were established to monitor all CHL data collection outcomes, but are not covered here.

## Results

The QA visits occurred between January 2013 to May 2015 across all jurisdictions. Alaska, American Samoa, CNMI, Guam, and Hawai‘i had two visits; baseline and follow-up. Baseline visits were held from January to December 2013 and follow-up visits from January to April 2015. FAS QA visits were held from October 2013 to May 2015.

The QA results per visit at baseline and follow-up are presented in Table 2. The majority of jurisdictions followed all of the “in the office” and “in the field” protocol standards. The 24 month follow-up visits showed an overall improvement in maintaining study protocols from baseline. The jurisdictions that participated in the 24-month measurement showed an improvement in QA scores from baseline to follow-up. The QA processes such as form storage, anthropometry/Acanthosis Nigricans station, forms station, and checkout station improved by 20 %. Consent and assent forms and accelerometer downloads and resetting improved by 40 %. Orientation/check-in went from one (10 %) jurisdiction meeting all protocols to five (100 %) meeting all protocols during 24-month follow-up. Accelerometer placement station decreased by 20 % from baseline to follow-up. Accelerometer log (100 %), completed forms/logs (100 %), food and activity log/accelerometer instruction (60 %), and transport of forms remained constant between baseline and follow-up. Orientation/check-in improved the most by one (20 %) to five (100 %) jurisdictions meeting all protocols.

**Table 2:** Baseline and follow-up results of the Children’s Healthy Living (CHL) program “in the office” and “in the field” quality assurance (QA) process

QA process	Prevalence sites		Intervention sites			
	Baseline^a^ (n = 11)		Baseline^b^ (n = 5)		Follow-up^b^ (n = 5)	
	All protocols met	Protocols not met	All protocols met	Protocols not met	All protocols met	Protocols not met
*In the office*						
Form storage	6 (55 %)	5 (45 %)	3 (60 %)	2 (40 %)	4 (80 %)	1 (20 %)
Consent and assent forms	5 (45 %)	6 (55 %)	2 (40 %)	3 (60 %)	4 (80 %)	1 (20 %)
Accelerometer log	11 (100 %)	0	5 (100 %)	0	5 (100 %)	0
Accelerometer downloads and resetting	9 (82 %)	2 (18 %)	3 (60 %)	2 (40 %)	5 (100 %)	0
Completed forms/logs	11 (100 %)	0	5 (100 %)	0	5 (100 %)	0
*In the field*						
Orientation/check-in	4 (36 %)	7 (64 %)	1 (20 %)	4 (80 %)	5 (100 %)	0
Food and activity log/accelerometer instruction	8 (73 %)	3 (27 %)	3(60 %)	2 (40 %)	3 (60 %)	2 (40 %)
Anthropometry/Acanthosis Nigricans station	3 (27 %)	8 (73 %)	1 (20 %)	4 (80 %)	2 (40 %)	3 (60 %)
Accelerometer placement station	8 (73 %)	3 (27 %)	3 (60 %)	2 (40 %)	2 (40 %)	3 (60 %)
Forms station	4 (36 %)	7 (64 %)	1 (20 %)	4 (80 %)	2 (40 %)	3 (60 %)
Check out station	7 (64 %)	4 (36 %)	3 (60 %)	2 (40 %)	4 (80 %)	1 (20 %)
Transport of forms	7 (64 %)	4 (36 %)	4 (80 %)	1 (20 %)	4 (80 %)	1 (20 %)

^a^Results include all 11 jurisdictions

^b^Baseline results among the five jurisdictions participating in the intervention study, for comparison with the 24 month follow-up measurements

## In the office

### Form storage

All jurisdictions stored forms securely in locked cabinets behind a locked door. During baseline, some jurisdictions initially stored the protected health information and non-protected health information files within the same cabinets. This was promptly corrected during the QA visit. At follow-up, the majority of jurisdictions met all protocols.

### Consent and assent forms

In certain jurisdictions, both consent and assent forms were not filed properly in participant folders or collected from parents/caregivers at the appropriate time. Follow-up corrected the error.

### Accelerometer log

All jurisdictions had all accelerometers in-house except for those placed in the field. Methods of organization differed by jurisdiction but were in place at all locations to ensure that accelerometers were not misplaced.

### Accelerometer downloading and resetting

The majority of jurisdictions downloaded and reset accelerometers correctly. The accelerometers are time sensitive and have a recommended time frame to download data. Some jurisdictions delayed downloading the data until after the QA visit which increased the amount of time it took to save the data, since those devices were still recording data during that time.

### Completed forms/logs

All jurisdictions had their forms/logs accounted for but experienced some common data quality problems with completing these forms/logs. Issues included not reviewing forms for thorough completion and not obtaining clarification from parents for unintelligible and illegible responses. For example, a question on the number of people in household did not equal the sum of the number individuals in household by age group, or the child’s age and parent provided birthdate did not coincide. Other points of misclassification included listing the child’s age when they were no longer breastfed in years instead of in months, the unit of time indicated for the response.

## In the field

In jurisdictions where languages other than English were used in the field, a native speaker assisted the CHL QA lead with explaining food and activity log instructions and accelerometer placements through translation and appropriate hand gestures. Forms, excluding food and activity log instructions, were provided in English and other native languages, which were translated by a native speaker.

### Orientation/check-in station

Most jurisdictions followed proper procedures for check-in at baseline and all at follow-up. Issues included ensuring that consent and photo release forms were signed and that the assent form of the child was collected before measurement.

### Food and activity log/accelerometer instruction station

The majority of jurisdictions followed proper procedures for food and activity log/accelerometer instruction. For food and activity log/accelerometer instructions, jurisdictions often did not emphasize recording the project’s high priority items such as fruits, vegetables, beverages, start and end times for activities, and activity description. There was an inconsistent use and demonstration of instructional tools such as measuring cups and spoons, sample wrappers and labels, labeled Ziploc bags, and recipes to parents. A commonly overlooked procedure was labeling food and activity logs with ID numbers.

### Anthropometry/Acanthosis Nigricans station

All jurisdictions completed assent forms prior to anthropometric measurement. Some jurisdictions did not calibrate equipment prior to every measurement session or attempt three measurements per child, according to protocol. Minor measurement procedures were overlooked during sessions such as removing children’s socks, hair ties, and heavy belts, viewing measurements at eye level, facing child away from scale screen, placing the tape measure horizontally around child’s waist, indicating diaper use, and measurement verification between measurer and recorder. Generally, Acanthosis Nigricans screening was properly followed while occasionally the staff failed to be discreet about observations for participant confidentiality or failed to ensure that all positive screens were verified.

### Accelerometer placement station

Overall proper procedures were followed at the accelerometer placement station for all jurisdictions. However, the use of safety scissors for cutting bands off children needed to be emphasized. Jurisdictions also needed to be reminded about letting the child choose their band color, placing ID labels onto correct logs, and reminding parents of the extra bands provided for the child.

### Forms station

All jurisdictions experienced problems with providing proper oversight at the forms station. Preparing reference material in advance for parents, designating an area for children to play while parents filled out forms, reviewing forms using different colored writing instruments from parents/caregivers, and being proactive in identifying parents who needed assistance prior to form disbursement were issues that needed to be addressed. CHL staff also needed to improve reviewing all forms and verifying completion prior to parent/caregiver’s departure. Jurisdictions were reminded to review forms station procedures and prepare forms properly prior to measurement sessions.

### Check out station

Many jurisdictions did not complete the participant folders’ check lists or complete necessary logs for tracking purposes at the end of measurement sessions, which were then corrected in the office. Organizing forms of protected health information and non-protected health information into separate folders was generally completed by all jurisdictions.

### Transport of forms and data

The majority of jurisdictions followed proper procedures for transport of forms with separate lockable repositories for protected health information and non-protected health information forms. All jurisdictions were reminded to review data transportation procedures to prevent protected health information classification breaches.

## Discussion

The purpose of this paper was to document the QA processes for conducting a multi-site childhood obesity prevalence survey and intervention trial across the USAP region. The implications of geographic location and diverse cultural settings were considered during the development of standard protocol procedures. Methods were revisited and adapted to the environment and culture while still meeting the standards of CHL, based on QA visit findings. For example, *locked boxes* were expanded to include locked backpacks or locked plastic containers (e.g., action packers). Some sites had limited resources, as such, specific survey items, such as locked boxes, were not readily available. Also, items such as locked boxes may bring unwanted attention to data collection personnel.

The increase in time for accelerometer download and resetting caused a delay for accelerometer use, which decreased the amount of children’s activity measured. To resolve this issue, each jurisdiction developed a schedule to download accelerometer data within three days. A CHL employee was designated to adhere to the schedule. Weekly data/measurement calls were also held with all jurisdictions to update accelerometer status as well as other data-related issues.

QA had to be staggered across jurisdictions due to great travel distances. In order to ensure consistency in feedback and reduce bias, CHL had QA site visits conducted by one individual. Each jurisdiction held a debriefing meeting after the QA assessment to discuss the team’s experience and solutions for protocols that were not met. Changes to the program’s procedures were made following the QA to correct the issues that occurred. Jurisdictions may have altered certain steps in collecting data to address the cultural factors within their respective communities. The demonstration of wearing an accelerometer by CHL staff was not required but proved effective in gaining a child’s assent to wear one. For a family with two or more children recruited, ID labels on accelerometer bands were used for parent identification. In cultural settings, native languages were used to talk with parents/caregivers and food examples were based on the jurisdiction’s usual diet. Verification of completed forms at every station was important to reduce the oversight of response errors. Parents/caregivers may not have answered all questions or provided complete answers. For example, for the question asking for the number of people living in the household, if the tally marks and total did not match, then the data entry software would recognize an error and prevent continuation of entry until the problem was resolved. Identifying and correcting this issue in the field proved beneficial to preventing future data entry problems.

The greatest strength of the CHL QA program was the standardized protocol used throughout the multi-site study that limited confounding bias. The high level of collaboration and efficiency with jurisdiction sites where local staff were hired and trained resulted in an organized, novel and functional recruitment process as outlined in Fialkowski et al. [26]. The QA site visit process was an important component to ensure data integrity within the multi-site study in this underserved, underreported and underrepresented region. Similar to other community-based studies such as the Girls Health Enrichment multi-site studies (GEMS) [17], the CHL QA approach addressed the complexity of implementing a study of this nature across 11 environmentally, geographically, and culturally distinct jurisdictions through flexibility and adaptation. This required significant time, resources, coordination and collaboration across a team of more than 100 individuals. Future research endeavors in similar environments such as the USAP wishing to maintain standardized protocols should consider these factors when developing their QA process.

More is needed in this area as the QA processes conducted in research studies related to multi-site and obesity prevention are limited. Unidentified systematic and non-systematic errors may have occurred from lack of measurement oversight and protocol neglect. However, sharing of research protocols, including QA protocols, through the peer-reviewed literature is an opportunity to prevent or mitigate potential systematic and non-systematic errors.

## Conclusions

Implementing a multi-site trial across a region as diverse and expansive as the USAP requires considerable effort to ensure consistency in data collection efforts. Although some procedures were overlooked in the office and in the field, corrections were made after the QA visit. This often included a review of study protocol. The reduction in errors at the 24-month QA visit for the intervention sites attests to the success of the program. The USAP is an under-studied area, and therefore experience with research methodology is limited. The QA process used in this region demonstrated that large community-based multi-site trials can be conducted in this unique geographic setting, following standardized yet community sensitive protocols. CHL provided a foundation for implementing a standardized research protocol that will serve as a framework for others to expand on. This paper provides a reference for others interested in conducting public health QA research in resource limited and geographically isolated locations.

## Abbreviations

CHL: Children’s Healthy Living Program
CNMI: Commonwealth of the Northern Mariana Islands
FAS: Freely Associated States of Micronesia
IRB: Institutional Review Board
QA: Quality Assurance
USAP: US Affiliated Pacific
